# Maternal low-protein diet reduces skeletal muscle protein synthesis and mass *via* Akt-mTOR pathway in adult rats

**DOI:** 10.3389/fnut.2022.947458

**Published:** 2022-08-30

**Authors:** Diogo Antonio Alves de Vasconcelos, Renato Tadeu Nachbar, Carlos Hermano Pinheiro, Cátia Lira do Amaral, Amanda Rabello Crisma, Kaio Fernando Vitzel, Phablo Abreu, Maria Isabel Alonso-Vale, Andressa Bolsoni Lopes, Adriano Bento-Santos, Filippe Falcão-Tebas, David Filipe de Santana, Elizabeth do Nascimento, Rui Curi, Tania Cristina Pithon-Curi, Sandro Massao Hirabara, Carol Góis Leandro

**Affiliations:** ^1^Department of Nutrition, Center of Health Sciences, Federal University of Pernambuco, Recife, Brazil; ^2^Post-graduate Program in Nutrition, Physical Activity and Phenotypic Plasticity, Federal University of Vitória de Santo Antão, Vitória de Santo Antão, Brazil; ^3^Department of Physiology and Biophysics, Institute of Biomedical Sciences, University of São Paulo, São Paulo, Brazil; ^4^Quebec Heart and Lung Institute Research Center, Laval University, Quebec City, QC, Canada; ^5^School of Health Sciences, College of Health, Massey University, Auckland, New Zealand; ^6^The Ritchie Centre, Hudson Institute of Medical Research, Department of Obstetrics and Gynaecology, Monash University, Melbourne, VIC, Australia; ^7^Interdisciplinary Post-graduate Program in Health Sciences, Cruzeiro do Sul University, São Paulo, Brazil

**Keywords:** low-protein diet, insulin resistance, protein metabolism, developmental plasticity, Akt expression

## Abstract

Several studies have demonstrated that a maternal low-protein diet induces long-term metabolic disorders, but the involved mechanisms are unclear. This study investigated the molecular effects of a low-protein diet during pregnancy and lactation on glucose and protein metabolism in soleus muscle isolated from adult male rats. Female rats were fed either a normal protein diet or low-protein diet during gestation and lactation. After weaning, all pups were fed a normal protein diet until the 210th day postpartum. In the 7th month of life, mass, contractile function, protein and glucose metabolism, and the Akt-mTOR pathway were measured in the soleus muscles of male pups. Dry weight and contractile function of soleus muscle in the low-protein diet group rats were found to be lower compared to the control group. Lipid synthesis was evaluated by measuring palmitate incorporation in white adipose tissue. Palmitate incorporation was higher in the white adipose tissue of the low-protein diet group. When incubated soleus muscles were stimulated with insulin, protein synthesis, total amino acid incorporation and free amino acid content, glucose incorporation and uptake, and glycogen synthesis were found to be reduced in low-protein diet group rats. Fasting glycemia was higher in the low-protein diet group. These metabolic changes were associated with a decrease in Akt and GSK-3β signaling responses to insulin and a reduction in RPS6 in the absence of the hormone. There was also notably lower expression of Akt in the isolated soleus muscle of low-protein diet group rats. This study is the first to demonstrate how maternal diet restriction can reduce skeletal muscle protein and mass by downregulating the Akt-mTOR pathway in adulthood.

## Graphical Abstract

Maternal low-protein diet reduces skeletal muscle protein synthesis and mass *via* the akt-mTOR pathway in adult rats. 1. Developmental plasticity determined by maternal low-protein during pregnancy and lactation in the skeletal muscle in the long term. 2. Downregulation of Akt expression signaling modulated by developmental plasticity; 3 and 4. Impaired Akt-mTOR pathway reduced insulin sensibility, free amino acids, and glycogen and protein synthesis. These molecular and metabolic changes produced a permanent phenotype, including insulin resistance associated with muscle atrophy in adulthood. Full arrows (black and red) indicate results obtained in the present study; dashed arrows indicate findings reported in other studies or that may occur in skeletal muscle; lines ending with a bar indicate inhibitory effects. 
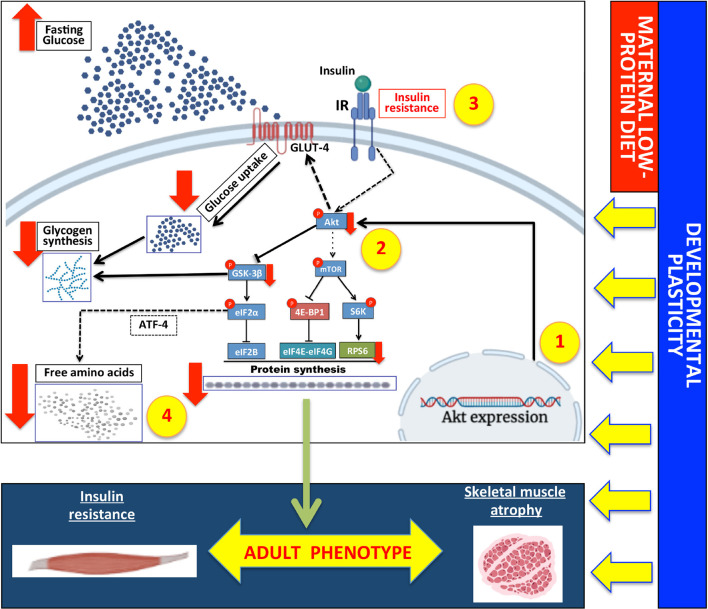

## Introduction

Epidemiological studies have reported an association between an early low-protein diet and the appearance of insulin resistance, type 2 diabetes, and metabolic disorders in adulthood (1, 2). The Developmental Origins of Health and Diseases (DOHaD) has focused on the importance of developmental plasticity during early life for health and disease (3). Ozanne et al. (4) found that a low protein diet during pregnancy and lactation caused a loss of soleus mass associated with impaired insulin signaling in adulthood. However, there is still a need for a detailed description of the protein metabolism changes associated with deficient insulin action in skeletal muscle induced by a maternal low-protein diet and their underlying molecular mechanisms. This may, in turn, lead to novel strategies for the treatment of related diseases, such as type 2 diabetes mellitus.

Skeletal muscle mass accounts for 40–50% of body weight and metabolizes about 80% of glucose under post-prandial conditions. Maintenance of muscle mass is in turn critical for glycemia homeostasis (5). An unbalanced diet during the early phase of life impairs protein activation and expression of insulin signaling in the long term (4, 6). Akt is part of insulin signaling pathway, particularly when phosphorylated at the serine 473 and threonine 308 residues, leading to the anabolic effects on the cells, including protein turnover and glucose metabolism (7–9). Previous studies have demonstrated that a maternal low-protein diet induces impairment in the Akt activation in both residues in the offspring (9), which has anabolic effects on the cells, including protein turnover and glucose metabolism. Its main effects include: (a) glucose uptake, by increasing GLUT-4 translocation from inner vesicles to the plasmatic membrane *via* PKCs (4, 10), followed by glucose molecules providing substrates for phosphorylation by hexokinase, forming glucose-6-phosphate; (b) phosphorylation of the GSK3-β leads to glycogen synthesis using glucose-6-phosphate resulting from glucose uptake (11), while the phosphorylated GSK3-β has a slight effect on protein translation *via* eIF2α (Initiation translation factor 2α) (12); and (c) activation of the mTOR1 pathway leads to protein synthesis *via* 4E-BP1 (binding protein for initiation translation factors)—eIF4E (initiation of protein translation), and RPS6 (ribosomal protein) (ribosome synthesis) (11–13); activated mTOR complex 1 plays a significant role in the regulation of protein metabolism in skeletal muscle (12, 14, 15). A previous study has shown that a maternal low-protein diet reduces the expression of the key proteins of the insulin pathway, including PKC-Zeta, p-110 beta, p-85alfa, and GLUT-4, in the skeletal muscles of adult humans (19 years old, vastus lateralis skeletal muscle) and rats (15 months old, soleus muscle), although in these cases, Akt remained unaltered (16). Some studies have also assessed the effects of a restricted protein diet on the mTOR1 pathway at an early age (17), but not over a long-term period.

Insulin is capable of stimulating the uptake of substrate elements essential for glucose storage (7) and protein synthesis by increasing gene expression of ATF-4 (a transcriptional factor to carry amino acids) in skeletal muscle cells (18). These molecular pathways regulate the nutritional status of cells, generating an increase in glucose and amino acid uptake, followed by the synthesis of glycogen and protein, respectively (5, 19). Reduced insulin sensibility in adulthood caused by a maternal low-protein diet may have molecular effects on the skeletal muscle, impairing the turnover of amino groups and glucose homeostasis. Insulin resistance is associated with impaired Akt activation on serine 473 in skeletal muscle cells, causing elevated quantities of glucose in the extracellular fluid (7, 8). The excess glucose not used by the skeletal muscle owing to reduced insulin sensibility may be utilized by the white adipose tissue, which stores it as triacylglycerol. This process is mediated by insulin, which promotes glucose uptake, *de novo* fatty acid synthesis, and lipogenesis. Glucose generates citrate acted upon by ATP-citrate lyase; citrate is converted into acetyl-CoA by citrate lyase; the acetyl-CoA carboxylase enzyme acts on acetyl-CoA yielding malonyl-CoA (Glucose can also be used to synthesize 3-glycerol phosphate from dihydroxiacetona phosphate by glycolysis, catalyzed by glycerol 3-phosphate dehydrogenase) (20, 21). As a consequence, increased hypertrophy of adipose cells is caused by maternal low-protein-diet in the long term (22).

A number of studies have shown that a low-protein diet during gestation and lactation is an important factor in long-term impairment of insulin action by way of developmental plasticity, which has been revealed to be one of the etiologies of type 2 diabetes (16, 23). Previous studies using animal models have shown that a maternal low-protein diet modulates protein expression and insulin signaling activity in skeletal muscle at various stages in life (16, 17, 24). It is likely that reduced activation of the Akt_*ser*473_ found in adults caused by a maternal low-protein diet may modulate mTOR signaling and thereby impair protein metabolism. A previous study found a reduced number of skeletal muscle cells and myotube expansion with no alteration in differentiation in 90-day-old pups submitted to a maternal low-protein diet, indicating a negative impact on protein quantity and turnover (25). Reduced glucose and amino acid uptake, and consequent glycogen and protein synthesis, lead to muscle mass loss, which, in turn, increases the risk of developing related diseases, including type 2 diabetes. To date, the relationship between poor insulin signaling and diminished protein synthesis in the muscle tissue has not been addressed. In a previous publication, we have shown that an early nutrient insult can modify gene expression of the glycolytic proteins over time, especially in oxidative muscle (26). The present study aimed to evaluate the long-term molecular effects of a maternal low-protein diet on glucose and protein metabolism in the soleus muscle of offspring in adulthood.

## Materials and methods

### Animals

Wistar rats were obtained from the Department of Physiology and Biophysics, Institute of Biomedical Sciences, University of São Paulo. Animals were maintained at 23 ± 2°C, under a light/dark cycle of 12/12 h, with free access to food (Nuvilab CR1, Nuvital Nutrientes Ltda, Curitiba, PR, Brazil) and water. The experimental procedures were carried out in accordance with the recommendations laid out in the Guide for Care and Use of Laboratory Animals of the Institute of Biomedical Sciences, University of São Paulo (experimental protocol number: 117; 93-02).

### Experimental design

**FIGURE 1:**
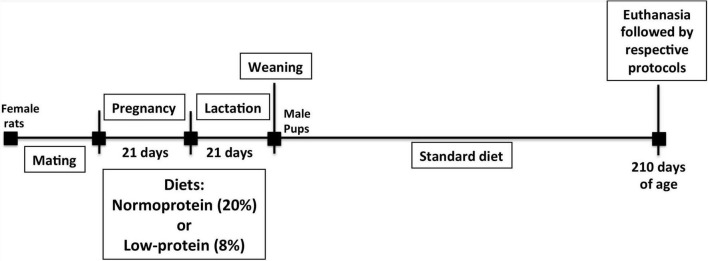
Experiment design. Once gestation was confirmed, a normoproteic or low-protein diet was administered to rats during pregnancy and lactation. After weaning, the offspring of both groups were fed a standard laboratory diet until the 210th day postpartum. The pups were then euthanized, and an analysis was performed.

Ten virgin female rats were mated randomly (2:1) at 60 days of age. The first day of gestation was confirmed when spermatozoids were found on histological slide analysis using a 16x microscope objective of vaginal secretion of female rats. Body weight was subsequently monitored. A normoproteic (20% of casein, *n* = 5) or low-protein (8% of casein, *n* = 5) diet was then administered to rats during pregnancy and lactation periods. When pups were born, the litter-size was established as eight pups per cage, two in each litter being required for different experiments. A total of 95 rats were therefore used. After the lactation phase, at 21 days postpartum, the offspring of both groups, normoproteic and low-protein (components of diet composition: Cornstarch, Casein (>85% protein), Dextrinized cornstarch (90–94% tetrasaccharides), Sucrose, Soybean oil (no additives), Fiber, Mineral mix (AIN-93G-MX), Vitamin mix (AIN-93-VX), L-Cystine, Choline bitartrate (41.1% choline), Tert-butylhydroquinone). Thus, the offsprings were weaned onto a standard laboratory diet (55% carbohydrate, 4.5% lipids, and 22.5% of protein was the amino acids source and contains: 3.5% L-glutamine, 1.7% L-leucine, 1.5% L-arginine, 1.2% L-lysine, 1.0% L-valine, 0.9% L-isoleucine, 0.8% threonine, 0.4% L-methionine, and 0.27% L-tryptophan. - Nuvilab CR1, Nuvital Nutrientes Ltda, Curitiba, PR, Brazil). Seven-month-old male pups from dams fed a normoproteic (control) or low-protein (LP) diet during pregnancy and lactation were used for all experiments (Figure 1). We have based this protocol in previous and classical studies that assessed the protein restriction during early life (4, 16). The body weight of dams was monitored during pregnancy and lactation and the pups were weighed on the 21st and 210th days using a Marte Scale (XL-500, II class) with 0.001 g precision. The mother’s food intake was determined as the difference between the amount of food (g) provided at 4 p.m. and the amount of food (g) that remained 24 h later. The results were normalized per 100 g of body weight.

### Analysis of the contractile function of skeletal muscle

The measurement of the contractile function of skeletal muscle was performed as previously described (27). The maximum force produced by the soleus muscle was evaluated by *in vivo* electrical stimulation. Animals were anesthetized with pentobarbital (75 mg/kg, i.p). Contractions were induced by electrical stimulation of the sciatic nerve at a voltage adjusted to produce the maximum force (around 10 V). Twitch force was determined by a stimulus consisting of 500 μs pulse duration at 1 Hz, while tetanic force was assessed at a frequency of 100 Hz. Data were recorded using AqDados (Version 4.16, Lynx Tecnologia Eletrônica Ltda, São Paulo, Brazil) and analyzed using AqAnalysis (Version 4.16, Lynx Tecnologia Eletrônica Ltda, São Paulo, Brazil). The animals used in this experimental protocol were independent of others due to high probability to change the physiological and molecular parameters.

### Protein synthesis rate and free amino acid content in isolated soleus muscle

After 3–4 h of fasting, rats were euthanized in a CO_2_ chamber and cervical dislocation was carried out. Soleus muscles were carefully excised and quickly isolated, and 25–35 mg strips were prepared and attached to steel clips to retain resting muscle tension. Muscles were then pre-incubated in a Krebs-Ringer bicarbonate buffer (KRBB), containing 5 mM glucose and 1% BSA, pH 7.4, at 35°C, for 30–40 min, at 100 oscillations per min. The protein synthesis rate was evaluated by incubating the muscle in Dulbecco’s Modified Eagle Medium (DMEM) containing 0.2 μCi/mL [U-^14^C] phenylalanine, under the same conditions, in the absence or presence of 7 nM of insulin, for 2 h. Throughout the pre-incubation and incubation periods, an atmosphere of 95% O_2_/5% CO_2_ was maintained. Muscle incorporation of [U-^14^C]phenylalanine into proteins (protein synthesis rate) and muscle-free [U-^14^C]phenylalanine (free amino acid content) were evaluated as previously described (28).

### Glucose metabolism in isolated soleus muscle

Muscles were then pre-incubated in a KRBB, containing 5 mM glucose and 1% BSA, pH 7.4, at 35°C, for 30–40 min, at 100 oscillations per min to ensure appropriate medium perfusion to the tissues. During all the incubation time, the medium was constantly maintained under 95% O_2_ and 5% CO_2_ supply. Phenylethylamine, diluted in methanol (1:1 v/v), was added by way of a microtube (0.3 mL) inside the incubation flask for ^14^CO_2_ adsorption. Incubation was performed for 1 h, under the same conditions, in the absence or presence of 7 nM insulin. After incubation, the muscles were processed to determine 2-deoxy-[2,6-^3^H]D-glucose uptake, [U-^14^C]D-glucose incorporation, [^14^C]-glycogen synthesis, and [U-^14^C]D-glucose decarboxylation, in accordance with the methods previously described and routinely performed by our group (7, 8).

### Western blot

Western blot assays were performed as previously described (5, 7, 8, 14). Soleus muscles were removed and pre-incubated as described above. The muscles were incubated in the absence or presence of 7 nM insulin for 45 min (7, 8). They were then homogenized in 10 mg/mL of lysis buffer containing proteases and phosphatase inhibitors. The antibodies used were anti-Akt Ser473 (#9271), anti-Akt (#9272), anti-GSK3β Ser9 (#5558), anti-GSK3β (#12456), anti-RPS6 Ser240/244 (#5364), anti- RPS6 (#2217), anti-4E-BP1 Thr37/46 (#2855), or anti-4E-BP1 (#9452). The secondary antibody was ANTI-RABBIT IGG, HRP-LINKED ANTIBODY (#7074S). All antibodies were purchased from Cell Signaling Technology. Results were normalized to total protein content as determined by Ponceau S staining and presented as arbitrary units as described in our previous studies (5, 7, 14). The whole lane, between 10 and 300 KDa for each sample, was analyzed in the Ponceau S stained membranes (Supplementary Figure 4).

### Statistical analysis

The GraphPad Prism 5 software (Graph Pad Software, Inc., San Diego, CA, United States) and G*Power program (Heinrich-Heine-Universität Düsseldorf, Düsseldorf, German) were used for statistical analysis and power analyses, respectively. Statistical differences were analyzed by unpaired *t*-test or two-way ANOVA followed by Bonferroni post-test. The α adopted for all protocols was 0.05.

## Results

### Maternal low-protein diet reduces soleus mass and impairs contractile function

**FIGURE 2:**
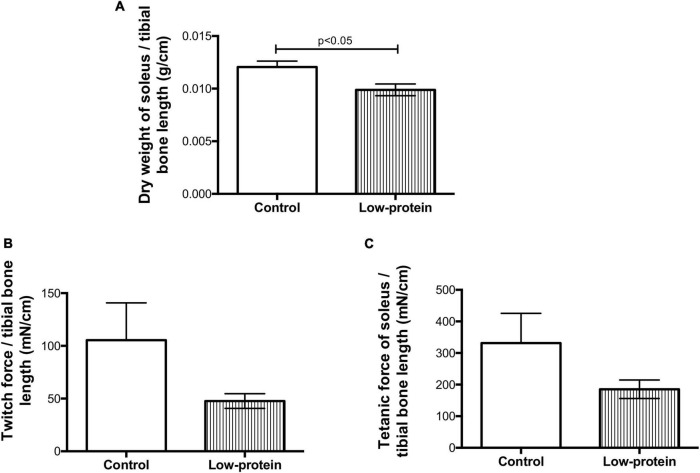
Long-term effects of low-protein diet during early life on mass and function of soleus muscle in rats. **(A)** Dry weight, **(B)** twitch force, and **(C)** tetanic force in soleus muscle of 7-month-old pups born to mothers fed a normoproteic (control, *n* = 6–10) or low-protein (LP, *n* = 6–10) diet during pregnancy and lactation. Results were analyzed using unpaired *t*-test. Values expressed as mean ± SEM.

The dry weight of soleus muscle from the low-protein diet group pups was lower (18%) when compared with those from the control group (Figure 2A). This reduction in muscle mass in the low-protein group pups is followed by skeletal muscle function to be reduced as well. Although it was not significant, the reduction in the percentage of twitch (Figure 2B) and tetanic (Figure 2C) forces were (54 and 44%, respectively).

### Long-term effects of maternal low-protein diet-induced on reduction in protein synthesis and quantity of amino acids in isolated soleus muscle

**FIGURE 3:**
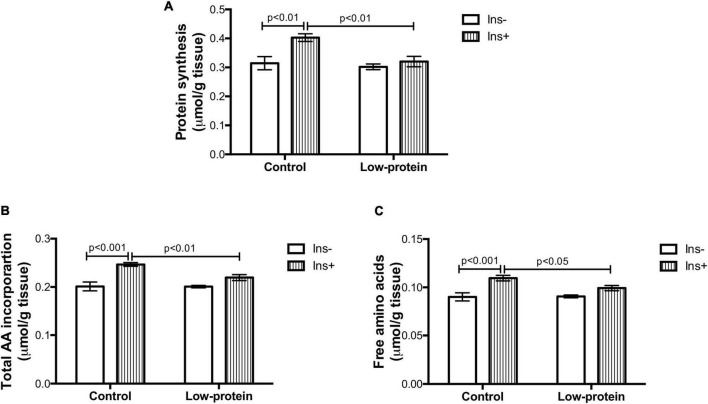
Long-term effects of low-protein diet during early life on protein metabolism in soleus muscle isolated from rats. **(A)** Protein synthesis rate, **(B)** total amino acid incorporation, and **(C)** free amino acids in isolated soleus muscle with or without insulin in 7-month-old pups from mothers fed a normoproteic (control, *n* = 6–8) or low-protein (LP, *n* = 6–8) diet during pregnancy and lactation. Results were analyzed using two-way ANOVA followed by Bonferroni post-test. Values expressed as mean ± SEM.

Insulin increased the protein synthesis rate (28%), incorporation of total amino acids (22%), and free amino acid content (21%) in the soleus muscle isolated from adult rats of the control group (Figures 3A–C). Insulin had no significant effect on any of these three parameters in the low-protein group, revealing an impairment of protein metabolism in response to the hormone (Figures 3A–C). The muscles from 7-month-old male rats born to low-protein-diet-fed mothers were less responsive to insulin than those of rats born to the control-diet-fed mothers, as evidenced by lower rates of protein synthesis (20%), total amino acid incorporation (11%), and free amino acid content (9%) (Figures 3A–C).

### Long-term maternal low-protein-diet-induced insulin resistance is associated with impaired glucose metabolism in isolated soleus muscle

**FIGURE 4:**
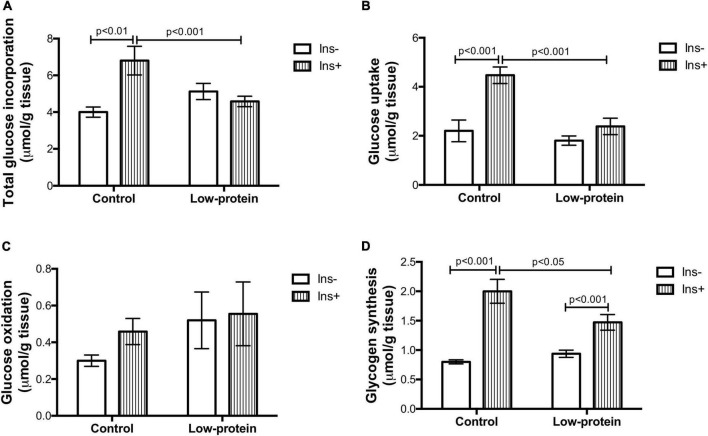
Long-term effects of low-protein diet during early life on glucose metabolism in soleus muscle isolated from rats. **(A)** Total glucose incorporation, **(B)** glucose uptake, **(C)** glucose oxidation, and **(D)** glycogen synthesis in isolated soleus muscle with or without insulin in 7-month-old pups born to mothers fed a normoproteic (Control, *n* = 6–8) or low-protein (LP, *n* = 6–8) diet during pregnancy and lactation. Results analyzed using two-way ANOVA followed by Bonferroni post-test. Values expressed as mean ± SEM.

Total incorporation of glucose and glucose uptake were increased by insulin in the soleus muscle in the control group (70 and 100%, respectively) (Figures 4A,B), while this was not observed in the low-protein groups. Both parameters were, however, much lower in low-protein group rats under insulin stimulation (glucose incorporation—32%; glucose uptake—46%). No changes were found in glucose oxidation in either group (Figure 4C). On the other hand, the insulin stimulus was able to increase glycogen synthesis in both groups (control-149%; LP-56%), but the increase was lower in the low-protein group than in the control group (26%) (Figure 4D).

### Long-term maternal low-protein-diet-induced downregulation of Akt-mTOR pathway in isolated soleus muscle

**FIGURE 5:**
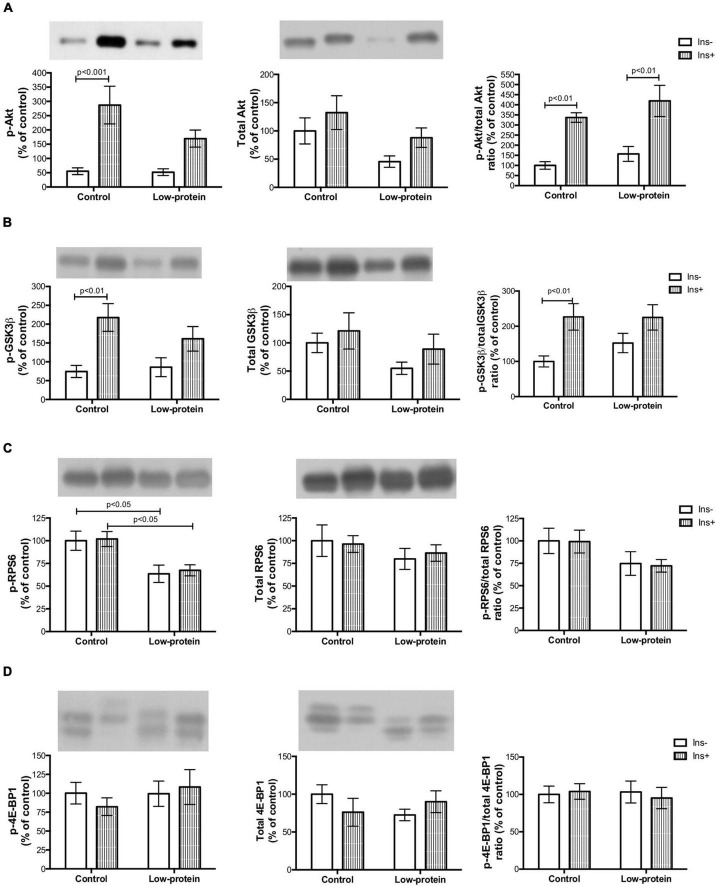
Long-term effects of low-protein diet during early life on AKt-mTOR pathway signaling in soleus muscle isolated from adult rats. **(A)** Akt Ser473, total Akt and Akt Ser473/Akt ratio; **(B)** GSK3β Ser9, total GSK3β and GSK3β Ser9/GSK3β ratio; **(C)** RPS6 Ser240/244, total RPS6 and RPS6 Ser240/244/RPS6 ratio; and **(D)** 4E-BP1 Thr37/46, total 4E-BP1 and 4E-BP1 Thr37/46/4E-BP1 ratio in isolated soleus muscle, with or without insulin, in 7-month-old pups born to mothers fed a normoproteic (control, *n* = 5–6) or low-protein (LP, *n* = 5–6) diet during pregnancy and lactation. Results were analyzed using two-way ANOVA followed by Bonferroni post-test. In **(A)**, *p* < 0.05 (Maternal diet effect) for LP groups (without and with insulin) compared to the respective control groups using two-way ANOVA only (no statistical differences using the Bonferroni post-test). All values are expressed as mean ± SEM.

Insulin increased Akt Ser473 (419%) (Figure 5A) and GSK3β Ser9 (192%) (Figure 5B) phosphorylation in the soleus muscle of offspring in the control group. There was, however, no statistical difference in Akt Ser473 and GSK3β Ser9 activation in the low-protein group (Figures 5A,B). We further observed that, in the low-protein diet group, there was a decrease in RPS6 Ser240/244 phosphorylation under both conditions (with and without insulin) when compared to the control (36%; 33%, respectively) (Figure 5C). While, there was no difference in 4E-BP1 (Figure 5D). It is worth noting that, with regard to protein expression, the present study found a statistical difference in total Akt only in the low-protein group (both with and without insulin stimulus) of 54 and 33%, respectively, compared to the control group (Figure 5A). Phosphorylated and total Akt ratio increased in both groups with insulin (control—236%; and low-protein—166%) (Figure 5A), whereas phosphorylated and total GSK3β was increased by insulin only in the control group (52%) (Figure 5B).

## Discussion

Ozanne et al. have reported that maternal protein restriction (8%) reduces soleus mass by 23%, when combined with disruption of insulin action, especially a reduction in PKCζ expression (4). The present study found that a maternal low-protein (8%) diet led to insulin resistance associated with impaired protein synthesis, affecting skeletal muscle mass and contraction activity (Figures 2, 3) in 7-month-old male rats. Moreover, a decrease in p-Akt and p-GSK-3β activation, and Akt downregulation in the skeletal muscle were found to be the molecular mechanisms relating to these phenotypes (Graphical Abstract).

Rats were fed a low-protein diet throughout pregnancy and lactation and then consumed a normal protein diet until 210 days of age. On postnatal day 21, low-protein diet rats weighed half as much as those in the control group. However, at 210 days of age, these animals achieved the same weight and length as the controls (Supplementary Figure 1). It is important to note that both groups were fed a normoproteic diet throughout life. Previous studies have shown that the catch-up growth associated with metabolic plasticity occurred in various tissues, including skeletal muscle and adipose tissue, thereby changing body composition (22, 29). The results of the present study thus suggest that, while insulin resistance (Figure 4) in the skeletal muscle of low-protein group adult rats caused an increase in fasting glucose (Supplementary Figure 2), the quantity of energy stored as triacylglycerol in white adipose tissue was augmented by the increase in lipid synthesis (Supplementary Figure 3). Detrimental long-term consequences were the result of a mismatch between perinatal time and weaning to adulthood (30). The data suggesting disturbances of glucose and protein metabolism (Figures 3, 4) and the disruption of the signaling pathways in the skeletal muscle corroborate the hypothesis regarding developmental plasticity and the underlying molecular mechanisms suggested by West-Eberhard (23).

A maternal low-protein diet brings about long-term changes in insulin signaling pathways in the skeletal muscle (4). Reduced Akt_*ser*473_ phosphorylation is a molecular marker of insulin resistance in this tissue (7, 8). Ozanne et al. (4) have revealed that a low-protein diet in the early phase causes decreased Akt activation and PKC ζ content in the soleus muscle of adult rats (4) but not in the expression of Akt protein, as shown by the present study. Reduced Akt expression in LP rats may have caused the low Akt activation found in the same group (Figure 5A). The developmental plasticity established for Akt expression may have affected the activation of downstream proteins of the mTOR pathway in the long term.

Reduced Akt activation by phosphorylation causes weak phosphorylation of the GSK-3β (involved in glycogen and protein synthesis) (Figure 5B) as previously observed by other studies (12). The mTOR1 is an important route leading to protein synthesis (12). However, we have found that the maternal low-protein diet group decreased RPS6 Ser240/244 phosphorylation without of insulin stimulus (Figure 5C). A limitation point of our study may be related to the incubation time used in our experimental protocol (45 min with insulin stimulation), since RPS6 activation is transitory and highly susceptible to dephosphorylation (31). One study assessing insulin resistance induced by a postnatal high-fat diet has also found alterations in the mTOR_1_ followed by reduced phosphorylation of Akt_*ser*473_ (32). Nevertheless, a maternal low-protein diet was able to change activation mTOR1 signaling (Figures 5C,D), which is one of the pathways that modulate protein synthesis (Figure 3), affecting both skeletal muscle mass and function (Figure 2).

A number of factors linked to the nutritional environment are effective in modulating protein metabolism. Failure of insulin action may modulate the metabolic environment of muscle cells (10). In fact, glucose and amino acid uptake, as well as protein and glycogen synthesis, were all found to be reduced (Figures 3, 4). The low availability of glucose and amino acid in the muscle cells causes a decline in the anabolic stimulus for glycogen and protein synthesis (8, 18). A reduced supply of substrates for anaplerotic reactions to glycolysis thus causes inhibition of the GSK-3β and mTOR_1_ (*via* AMPK) pathways involved in the synthesis of carbohydrate and amino acid polymers (33). It should be noted that glycogen synthesis in the low-protein insulin-treated group was lower and that the incorporation of glucose in LP muscles stimulated by insulin was similar to the incorporation of amino acids and free amino acids (Figures 3B,C, 4A). Even so, the glycogen in this group was probably synthetized by insulin stimulation using only a small quantity of amino acids and glucose as substrates. Low levels of glucose and amino acids were therefore probably sufficient to accumulate glycogen (Figure 3), but not protein. We also observed that GSK-3β phosphorylation is proportionally associated with a reduction in glycogen synthesis (Figures 4D, 5B). However, mTOR1 signaling, rather than activation of GSK-3β *via* eiF2α, is in fact the main controlling pathway of protein synthesis (12).

Impaired insulin sensibility influences anabolic effects, including the transport of essential metabolic precursors (amino acids and glucose). In relation to amino acid transportation, reduced amino acid importation occurred due to poor activation of the insulin pathway, probably due to ATF-4 up-regulation in the skeletal muscle cells (18). Glucose uptake was thus also found to be reduced throughout the AKT-PKC ζ pathway (4).

An excess of glucose in blood plasma finds its way into other tissues and is stored mainly in white adipose tissue (20, 21). Excess glucose cannot enter skeletal muscle, owing to the reduced insulin sensibility, and may therefore end up in white adipose tissue, where it can be used for triacylglycerol synthesis, a process mediated by insulin in several stages, including glucose uptake, fatty acid synthesis *de novo*, and lipogenesis (21). The white adipose tissue in the early low-protein rats thus independently presented high glucose uptake and fatty acid synthesis stimulated by insulin (preliminary data), as well as elevated lipogenesis (Supplementary Figure 3).

Restricted capacity to assess other metabolic tissues was one limitation of this study and it is for this reason that only a small number of experiments were conducted. On the other hand, one strength of the present study was the use of incubation to analyze both skeletal muscle and white adipose tissue. As the study of developmental plasticity involved long-term effects, the *in vitro* methodology was optimal in so far as it removed acute effects caused by the maternal diet producing alterations in physiological factors, such as the action of insulin on muscle being diminished by serum levels of glucocorticoids or fatty acids (34).

The main finding of the present study concerns the downregulation of Akt expression and Akt-mTOR signaling (Figure 5A) induced by a low-protein diet during the early phase, which was able to cause such a phonotype in skeletal muscle (Graphical Abstract). Long-term changes in gene expression may be regulated by epigenetic mechanisms, such as DNA methylation, histone modification, and non-coding RNAs (35, 36). It is likely that developmental plasticity was determined by inadequate nutritional stimulus during early life through epigenetic modulation regulating protein expression in the skeletal muscle in the long term (35, 37, 38). Our study is the first to demonstrate that a maternal diet leads, by way of the Akt-mTOR pathway, to insulin resistance with reduced skeletal muscle protein and mass in adulthood (Graphical Abstract). Our study thus suggests that the precise quantity of protein required during pregnancy and lactation is a subject that requires further elucidation, if we are to understand better how to avoid long-term metabolic disturbances that are strongly associated with obesity and Type 2 diabetes. Future studies are required to describe the underlying epigenetic regulation caused by early nutrition and thereby contribute to the prevention and treatment of related metabolic diseases.

## Data availability statement

The original contributions presented in this study are included in the article/Supplementary material, further inquiries can be directed to the corresponding author.

## Ethics statement

The experimental procedures were carried out in accordance with the recommendations laid out in the Guide for Care and Use of Laboratory Animals of the Institute of Biomedical Sciences, University of São Paulo (experimental protocol number: 117; 93-02).

## Author contributions

DAAV, RC, TCP-C, SMH, and CGL: research design. DAAV, RTN, KFV, SMH, and CGL: data analyses. DAAV, RTN, CHP, CLA, ARC, KFV, PA, MIA-V, ABL, AB-S, and SMH: research. DAAV, SMH, and CGL: wrote the manuscript. DAAV, RTN, CHP, CLA, ARC, KFV, PA, DFS, AB-S, FF-T, EN, RC, TCP-C, SMH, and CGL: revision of results and manuscript content. SMH and CGL: supervision. All authors contributed to the article and approved the submitted version.
