# Supplementary material for: Maternal low-protein diet reduces skeletal muscle protein synthesis and mass via Akt-mTOR pathway in adult rats
Source: Front Nutr. 2022 Aug 30;9:947458. doi: 10.3389/fnut.2022.947458 (PMC9468266; doi:10.3389/fnut.2022.947458)
Supplement: Supplementary file 1 [file Presentation_1.pdf]

## Supplemental information

# Maternal low-protein diet reduces skeletal muscle protein synthesis and mass via akt-mTOR pathway of adult rats

Diogo Antonio Alves de Vasconcelos[1,2,4]*, Renato Tadeu Nachbar[7], Carlos Hermano Justa Pinheiro[4], Cátia Lira do Amaral[4], Amanda Rabello Crisma[4], Kaio Fernando Vitzel[4,5], Phablo Abreu[4], Maria Isabel Alonso Vale[4], Andressa Bolsoni Lopes[4], Adriano Bento-Santos[2], Filippe Falcão-Tebas[6], David Filipe de Santana[2], Elizabeth do Nascimento[1], Rui Curi[3,4], Tania Cristina Pithon-Curi[3], Sandro Massao Hirabara[3], Carol Góis Leandro[2]

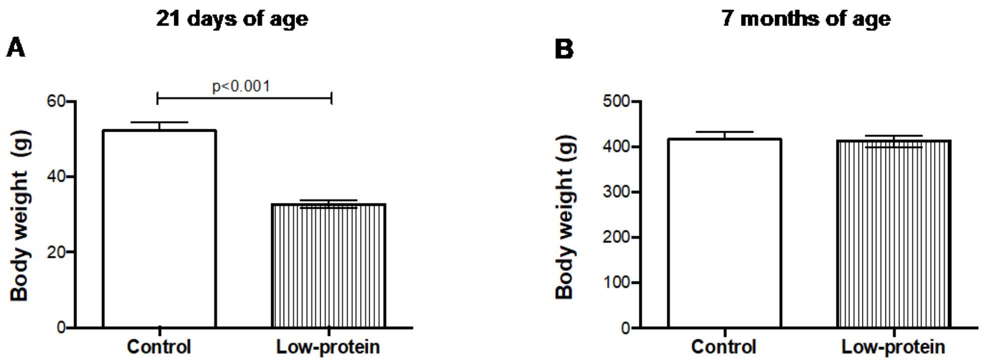


**Figure S1. Body weight at 21 days postpartum and at the age of 7 months of pups born to mothers fed a low-protein diet during pregnancy and lactation.** Body weight at 21 days (A) and at 7 months (B) of pups born to mothers fed a normoproteic (control, n=9-15) or low-protein (LP, n=9-15) diet during pregnancy and lactation. Results analyzed using an unpaired t test. Values expressed as mean± SEM.

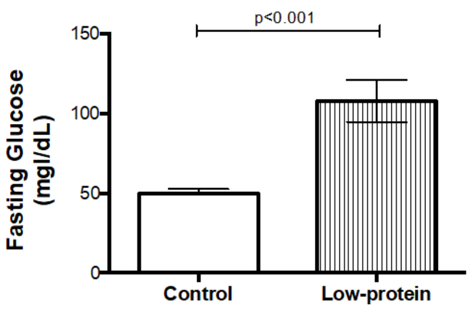


**Figure S2. Long-term effects induced by low-protein during early life on glycemia from adults rats.** The serum glucose concentration was determined using a commercial kit (glycemia - Laborclin, Pinhais, PR, Brazil) after 12 hours of fasting from rats at 7 months of age. $p<0.001$ for comparison between low-protein and control (unpaired t test). Values are expressed as mean ± SEM of 10-14 rats.

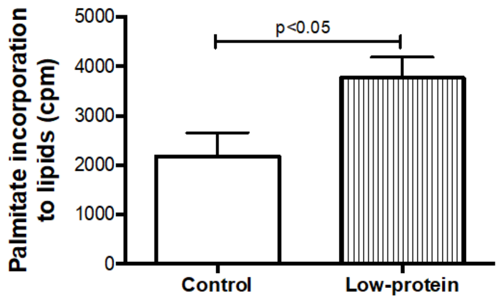


**Figure S3. Long-term effects induced by low-protein during early life on lipogenesis of white adipose tissue from adults rats.** The lipogenesis was determinate by [1-$^{14}$C]-palmitate incorporation into TAG, in isolated adipocytes extracted from epididymal adipose tissue of rats at 7 months of age. $p<0.05$ for comparison between low-protein and control (unpaired t test). Values are expressed as mean ± SEM of 4 rats.

A

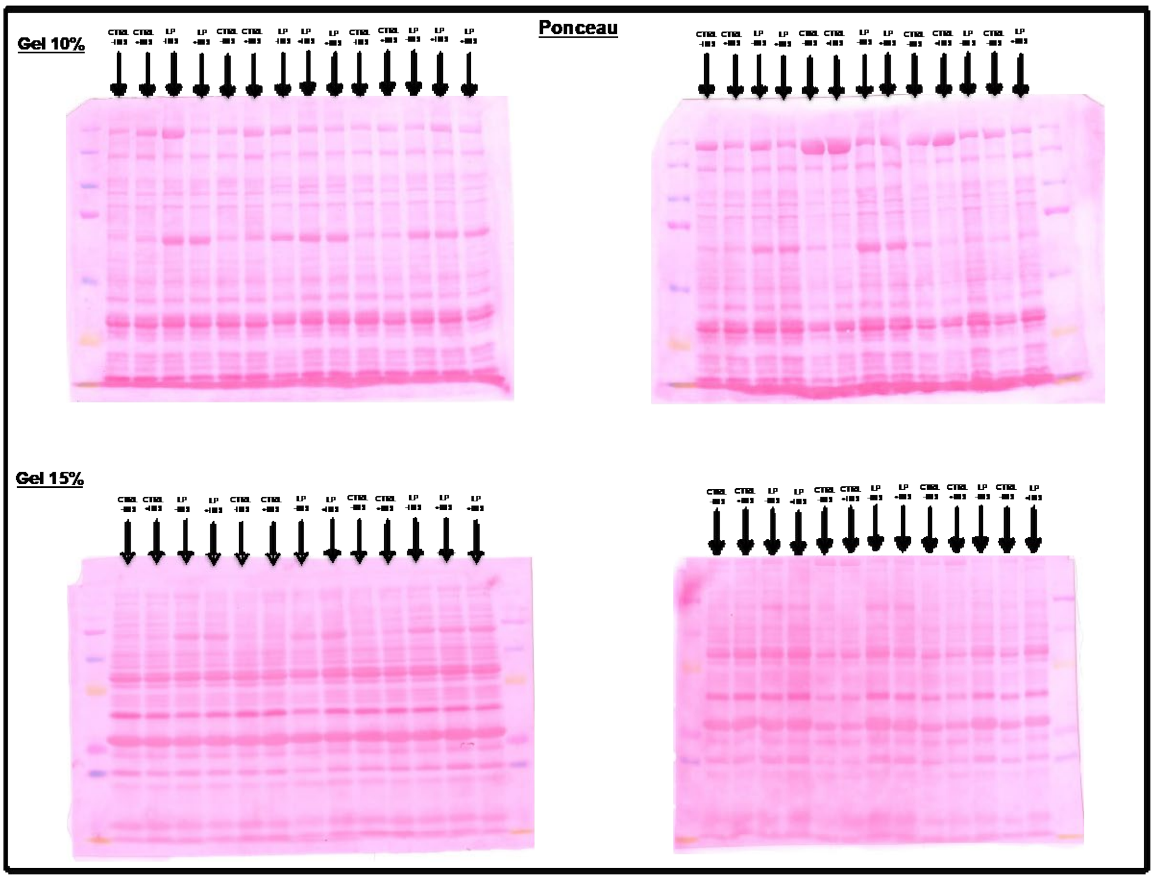
Ponceau
Gel 10%
Gel 15%

B

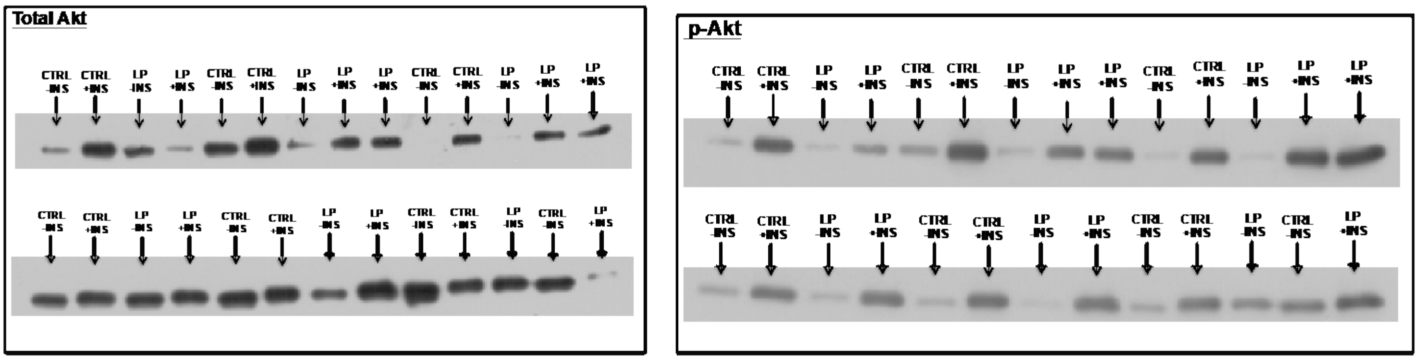
Total Akt
p-Akt

C

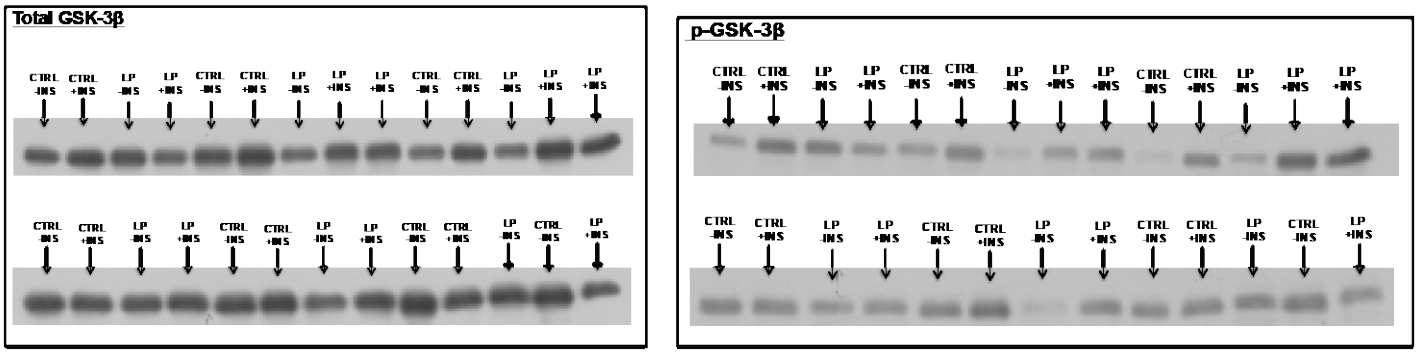
Total GSK-3β
p-GSK-3β

D

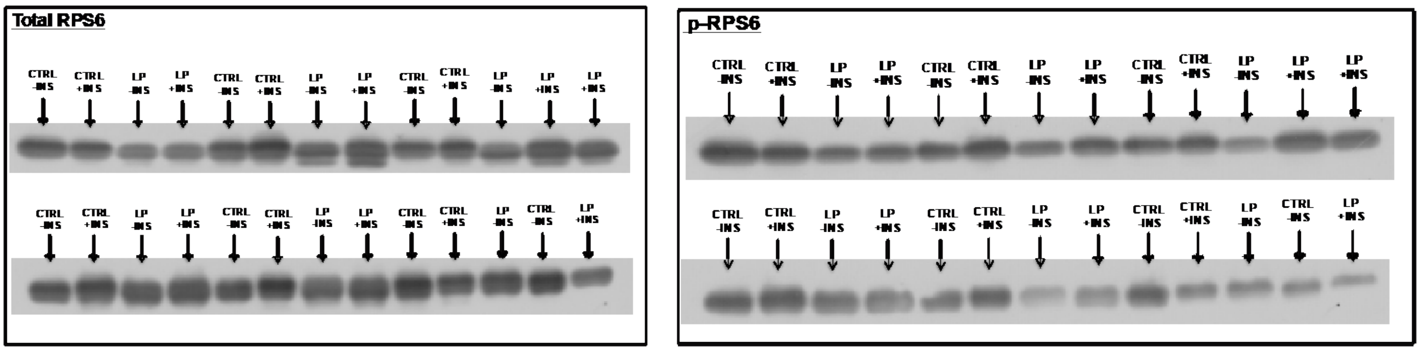
Total RPS6
p-RPS6

E

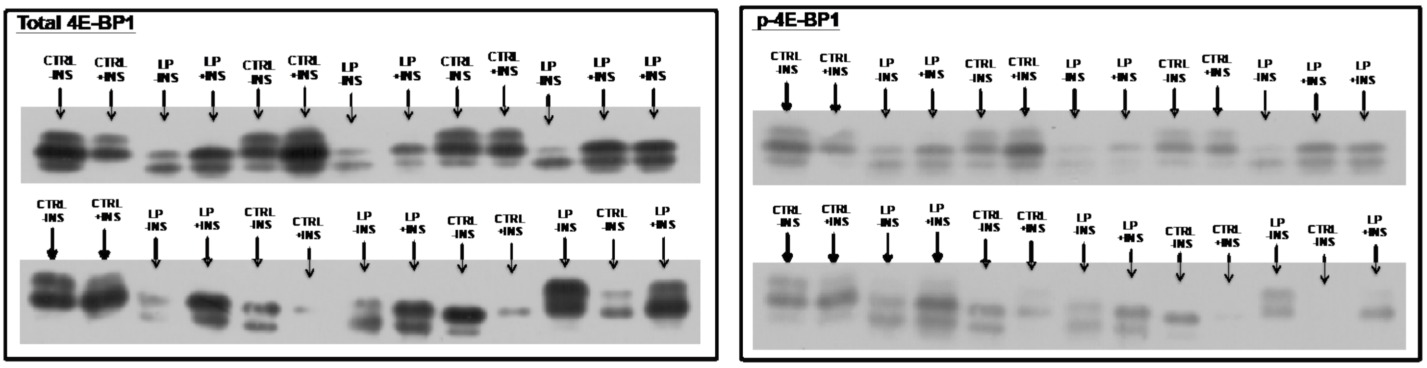


**Figure S4. Membranes and blots of western blotting assays of insulin signaling proteins in the incubated soleus muscle from adult rats submitted to the early low-protein diet.** Ponceau S staining of nitrocellulose membranes after protein transfer from 10% and 15% polyacrylamide gels (A); blots of total and phosphorylated Akt (B), GSK-3β (C), RPS6 (D) and 4E-BP1 (E).
